# Detection of localized pulsatile motion in cutaneous microcirculation by speckle decorrelation optical coherence tomography angiography

**DOI:** 10.1117/1.JBO.25.9.095004

**Published:** 2020-09-15

**Authors:** Peijun Gong, Christian Heiss, Danuta M. Sampson, Qiang Wang, Zhihong Yuan, David D. Sampson

**Affiliations:** a The University of Western Australia, Optical+Biomedical Engineering Laboratory, Department of Electrical, Electronic, and Computer Engineering, Perth, Western Australia, Australia; b The University of Surrey, Department of Clinical and Experimental Medicine, Faculty of Health and Medical Sciences, Guildford, Surrey, United Kingdom; c Surrey and Sussex Healthcare NHS Trust, Redhill, United Kingdom; d The University of Surrey, Centre for Vision, Speech, and Signal Processing, Surrey Biophotonics, Guildford, Surrey, United Kingdom; e The University of Surrey, School of Biosciences and Medicine, Surrey Biophotonics, Guildford, Surrey, United Kingdom; f The University of Surrey, Advanced Technology Institute, School of Physics, Surrey Biophotonics, Guildford, Surrey, United Kingdom

**Keywords:** optical coherence tomography angiography, speckle decorrelation, pulsatility, pulsatile motion, pulsatile blood flow, pulsatile pressure, cutaneous microcirculation

## Abstract

**Significance:** Pulsatility is a vital characteristic of the cardiovascular system. Characterization of the pulsatility pattern locally in the peripheral microvasculature is currently not readily available and would provide an additional source of information, which may prove important in understanding the pathophysiology of arterial stiffening, vascular ageing, and their linkage with cardiovascular disease development.

**Aim:** We aim to confirm the suitability of speckle decorrelation optical coherence tomography angiography (OCTA) under various noncontact/contact scanning protocols for the visualization of pulsatility patterns in vessel-free tissue and in the microvasculature of peripheral human skin.

**Results:** Results from five healthy subjects show distinct pulsatile patterns both in vessel-free tissue with either noncontact or contact imaging and in individual microvessels with contact imaging. Respectively, these patterns are likely caused by the pulsatile pressure and pulsatile blood flow. The pulse rates show good agreement with those from pulse oximetry, confirming that the pulsatile signatures reflect pulsatile hemodynamics.

**Conclusions:** This study demonstrates the potential of speckle decorrelation OCTA for measuring localized peripheral cutaneous pulsatility and defines scanning protocols necessary to undertake such measurements. Noncontact imaging should be used for the study of pulsatility in vessel-free tissue and contact imaging with strong mechanical coupling in individual microvessels. Further studies of microcirculation based upon this method and protocols are warranted.

## 1 Introduction

The pulsatile nature of blood flow is an important aspect of the cardiovascular system. Cardiovascular hemodynamics originates from the beating heart, which generates cyclic (pulsatile) blood pressure and flow. The ejected blood and pulse wave travel along the vascular tree. Importantly, the pulse wave travels faster than the blood itself and is reflected as it propagates.^1^ The reflected pulse wave interacts with the forward wave such that, depending on its velocity, the pressure may be increased locally, a phenomenon called augmentation. Because pressure and flow are inextricably linked in this context, these pulsatile hemodynamics may be referred to more generally as “pulsatility.” Pulsatility from the heart (initiated by ventricle contraction) passes through the large arteries and gradually attenuates toward the small arteries and the microvascular network, due to the cushioning (dissipative) function of the arterial tree.^2^ However, when stiffening of the arteries, a significant contributor to cardiovascular diseases, is present, the pulse wave travels faster and the cushioning function of the arteries is reduced. This greatly influences the pulsatility pattern (i.e., the time sequence of flow) even in the peripheral microcirculation.^3^^,^^4^

Measurement of the pulsatility pattern has been used to assess arterial stiffening in the context of important pathologies, such as systolic hypertension, heart failure, stroke, coronary artery disease, and diabetes mellitus.^5^ Due to the technical limitations of the current technology, pulsatility of the microcirculation cannot readily be measured and is, thus, not well characterized and relatively poorly understood. A few papers suggest that the reduced cushioning of the arterial tree can extend the pulsation further toward the microvasculature, leading to increased pulsation (and potentially damage) in the microvessels, such as in the brain and kidney.^6^ A large-scale study by Mitchell et al.^3^ reported the association of aortic stiffness with the peripheral microvascular function. Cooper et al.^4^ further highlighted the microvascular damage and remodeling as a pathway to mediate the associations between aortic stiffness and cardiovascular diseases. Therefore, measurement of the pulsatility locally in the peripheral microvasculature, ideally with the capability to map the network being measured, may provide an additional important source of information to understand the pathophysiology of arterial stiffening and vascular ageing, and to link these factors with cardiovascular disease development.

There are multiple methods to measure pulsatility, such as: sphygmomanometry, which measures the systolic and diastolic brachial artery pressure; electrocardiography, which uses electrodes to measure the electric current generated by the contracting heart muscle; and applanation tonometry, which records a pressure waveform through the skin above accessible superficial large arteries.^5^^,^^7^^,^^8^ Methods developed for measuring the blood flow have demonstrated the capability to measure pulsatile flow, such as those based on magnetic resonance imaging and Doppler ultrasonography.^9^^,^^10^ Although these methods have provided important insights into pulsatility, they target the relatively large vessels or large tissue volumes and are not easily adaptable to the peripheral microvascular network. There are also optical methods suited to peripheral measurements, such as laser Doppler flowmetry, photoplethysmography (i.e., pulse oximetry, which uses light to detect the blood volume changes), and other methods such as using the pulsatility induced displacement of the tissue surface.^11^^–^^14^ None of these methods is capable of visualization of the vessel network to enable characterization of pulsatility in a volumetric vessel-by-vessel context.

Optical coherence tomography angiography (OCTA) is a rapidly advancing method for imaging blood microvasculature (i.e., arterioles, capillaries, and venules) *in vivo*. Although largely investigated in the retina,^15^ OCTA also shows considerable promise in dermatology and neuroscience.^16^^–^^20^ It is a functional extension of optical coherence tomography (OCT), which measures tissue reflectance in three dimensions with micrometer-scale (1 to 20  μm) resolution. The vessel contrast in OCTA originates from the intrinsic motion of scatterers in blood vessels (mainly flowing blood cells), which induce readily detectable dynamic fluctuations in the OCT signal. In the static tissue surrounding the vessels, there are markedly lower fluctuations that are not due to blood flow but may be due to tissue displacement during heartbeats, and therefore, reflect the pulse pressure wave. Such fluctuations are captured by acquiring colocated OCT A-scans or B-scans from the same tissue locations for subsequent analysis with an algorithm to highlight the blood vessels. Multiple algorithms have been developed for OCTA, analyzing the fluctuations in the OCT complex signal [e.g., complex variance and optical microangiography (OMAG)], the intensity signal (e.g., speckle variance and speckle decorrelation/correlation mapping), or the phase signal (e.g., Doppler OCT and phase variance).^15^^,^^21^^–^^25^ Among them, Doppler OCT methods can measure the flow velocity component in the beam propagation (axial) direction and have been used in multiple early studies to observe pulsatile blood flow in microvessels.^26^^–^^34^ In contrast, other OCTA variants have not been adapted for pulsatility measurement, but have been widely used to visualize the vessel network, especially for commercially available OCTA retinal scanners. As a step in extending these variants for measuring pulsatility, a preliminary study by Xie et al. has recently explored the potential of the OMAG method.^35^

In this paper, we demonstrate that the widely used speckle decorrelation OCTA is capable of visualization of the microvascular pulsatility in the peripheral circulation in human skin and shows good agreement with pulse oximetry. We demonstrate characterization of pulsatility both in vessel-free tissue and in single microvessels, using both noncontact and contact scanning protocols. The pulsatile pattern in vessel-free tissue is reasonably expected to be caused by the pulsatile pressure wave; whereas, the pulsatile pattern in single microvessels is caused by pulsatile blood flow, with both potentially providing important complementary information in the context of cardiovascular pathophysiology, where arteriosclerosis (pulse wave), atherosclerosis (perfusion deficit by limited inflow), and microvascular disease are the main drivers of disease severity.

## 2 Methods

### 2.1 Study Subjects

Healthy subjects (n=5) were recruited for imaging with the ethics approval of the Human Research Ethics Committee at The University of Western Australia. Written consent was acquired from each subject prior to OCT scanning. The ages of the subjects ranged from 23 to 38 years (30±6  years).

### 2.2 OCT Imaging and Scanning Protocols

A commercial spectral-domain OCT scanner (upgraded TELESTO II, Thorlabs Inc., Newton, New Jersey), as previously described,^36^ was used in this study. In brief, the scanner provides an axial and lateral imaging resolution of, respectively, 5.5  μm (in air) and 13  μm (as specified by the vendor) at a center wavelength of 1300 nm and images at a raw line-scan rate of 76 kHz.

A noncontact and two contact scanning protocols were assessed for their capacity to demonstrate pulsatile motion in vessel-free tissue and in individual microvessels. These protocols were tested on the finger and forearm skin of the subjects, as summarized in Table 1. In addition, a pulse oximeter (Heart Sure a320, Shanghai International Holding Corp. Gmbh, Hamburg, Germany) was mounted on the contralateral middle finger to simultaneously monitor the heart rate during OCT scanning. The pulse oximeter displayed, but did not record, the pulsatile profiles; thus, the comparison of pulsatile profiles between speckle decorrelation and pulse oximeter measurements was performed by manually recording the average pulse rate from the pulse oximeter during the OCT scanning time.

**Table 1:** Summary of OCT pulsatility imaging of human skin.

Measurement set up and parameters			Pulsatile motion in vessel-free tissue	Pulsatile motion in microvessels
Scanning mode			Volumetric	M-mode
Skin location			Dorsal finger (adjacent to proximal nail fold); forearm	Forearm
Imaging protocol			Noncontact; contact with and without additional mechanical coupling	Contact with additional mechanical coupling
Scanning dimension	x direction	mm^a^	6 or 5	1.5
		pixels	1024	256
	y direction	mm^a^	4.5 or 3	—
		pixels	600	—
	z direction	mm	3.6	3.6
		pixels	1024	1024
Number of repeated B-scans per y location			2	1000
Total number of B-scans			1200	1000
Total scan time s^b^			21.4	7.7

a Scanning field of view of 5 mm×3 mm (x×y) was used for finger skin if excessive skin curvature resulted in low OCT signal at the edges of the 6 mm×4.5 mm field of view. Otherwise, 6 mm×4.5 mm was used.

b The scanning time was longer than the time estimated based on the scanning speed and the total number of acquired A-scans due to the additional time required for calibration in the OCT scanner.

#### 2.2.1 Imaging pulsatility in vessel-free tissue

A range of volumetric scanning procedures previously used for OCTA microvascular network imaging were applied to forearm and finger skin to test their applicability for detection of pulsatile motion in vessel-free tissue. Noncontact and contact imaging protocols were applied and compared in the following order: noncontact imaging under ideal conditions, noncontact imaging in the presence of confounding motion artifacts, contact imaging without additional mechanical coupling, and contact imaging with additional mechanical coupling (to be described later). Although noncontact imaging is more desirable in clinical applications, contact scanning with and without additional mechanical coupling has been previously used to improve OCTA image quality by mitigating extraneous motion.^18^^,^^25^

In noncontact imaging, the OCT probe was mounted on a holder and located above the skin. The ideal condition for noncontact imaging is when pulsatile motion caused by the pulsatile pressure wave is the dominant tissue motion.

In contact imaging without additional mechanical coupling, the probe mounted on a holder was gently placed in contact with the skin surface. Ultrasound gel was applied as an index-matching liquid, which has been shown to offer a range of imaging advantages and artifact mitigations.^37^

Contact imaging with additional mechanical coupling uses a previously developed strong mechanical coupling interface.^18^ In brief, the probe is mounted on an articulating arm and then gently handheld on the skin surface with contacting mechanical coupling. However, now a customized imaging spacer is used to adjust the distance between the OCT probe and the skin surface and the OCT probe with this spacer is attached to the skin with double-sided adhesive tape to further reduce the tissue motion relative to the OCT probe. The imaging set up also involves the attachment of a metal marker with a center hole (diameter: 5 mm). The marker allowed the examination of motion artifacts using the edges of the hole as well as enabled the application of ultrasound gel for index matching.

For both noncontact and contact imaging, we used the volumetric scanning protocol summarized in Table 1. Volumetric imaging was chosen as it enabled simultaneous visualization of the microvessel network in the imaging volume provided by standard OCTA processing. Two colocated OCT B-scans were acquired at each lateral location across the whole volume. A total of 14 volume scans were acquired from the five subjects.

#### 2.2.2 Imaging pulsatility in individual microvessels

The measurement of pulsatility in individual microvessels was not feasible with the volumetric scanning protocol due to the incompatibility between the requirement on sufficiently fast sampling of the individual vessels in order to observe their decorrelation and the volume acquisition rate (i.e., 21.4 s per volume). In addition, accurate location of the individual vessels over a long scanning time was not feasible with noncontact or contact imaging without additional mechanical coupling due to tissue motion. Therefore, for detection of pulsatility in single vessels, we applied contact imaging with additional mechanical coupling and M-mode scanning (Table 1). The lateral location for M-mode scanning was selected by first performing volumetric OCTA imaging to generate a quick preview of the vessel network and then locating the coordinates of the vessels of interest for M-mode scanning. Limited by the relatively large dimensions of the mechanical coupling device (with ∼30-mm-diameter contact surface), only the forearm was scanned. 12 M-mode OCT scans were acquired from the five subjects.

### 2.3 Data Analysis

#### 2.3.1 Analysis of pulsatility in vessel-free tissue

The acquired volumetric OCT scans were first processed using the speckle decorrelation algorithm to generate the corresponding OCTA volumetric scans. The algorithm was used to calculate the decorrelation of the OCT signal (i.e., amplitude of the complex OCT signal) between each pair of colocated B-scans across the whole volume, as previously described.^18^ The resulting OCTA scans show the desired high decorrelation in vessels, and in the static tissue, depending on the scanning protocol. Sources of motion other than those we seek to characterize can also lead to unwanted high decorrelation regardless of whether vessels or static tissue are being imaged, as do regions in which noise dominates. To remove the unwanted high decorrelation in regions with only OCT noise, the OCTA signal was weighted by the corresponding thresholded logarithmic OCT signal, with the threshold set by trial and error. The tissue surface was then segmented in the OCT scan using a Canny edge detector for subsequent extraction of the en face vessel projection images in the weighted OCTA volume.^38^ For visualization of the blood vessels, a maximum intensity projection (MIP) of the weighted OCTA signal was taken from the skin surface to typically 400 to 500  μm into the tissue, with the exact depth selected manually and specified in each case.

**Fig. 1:**
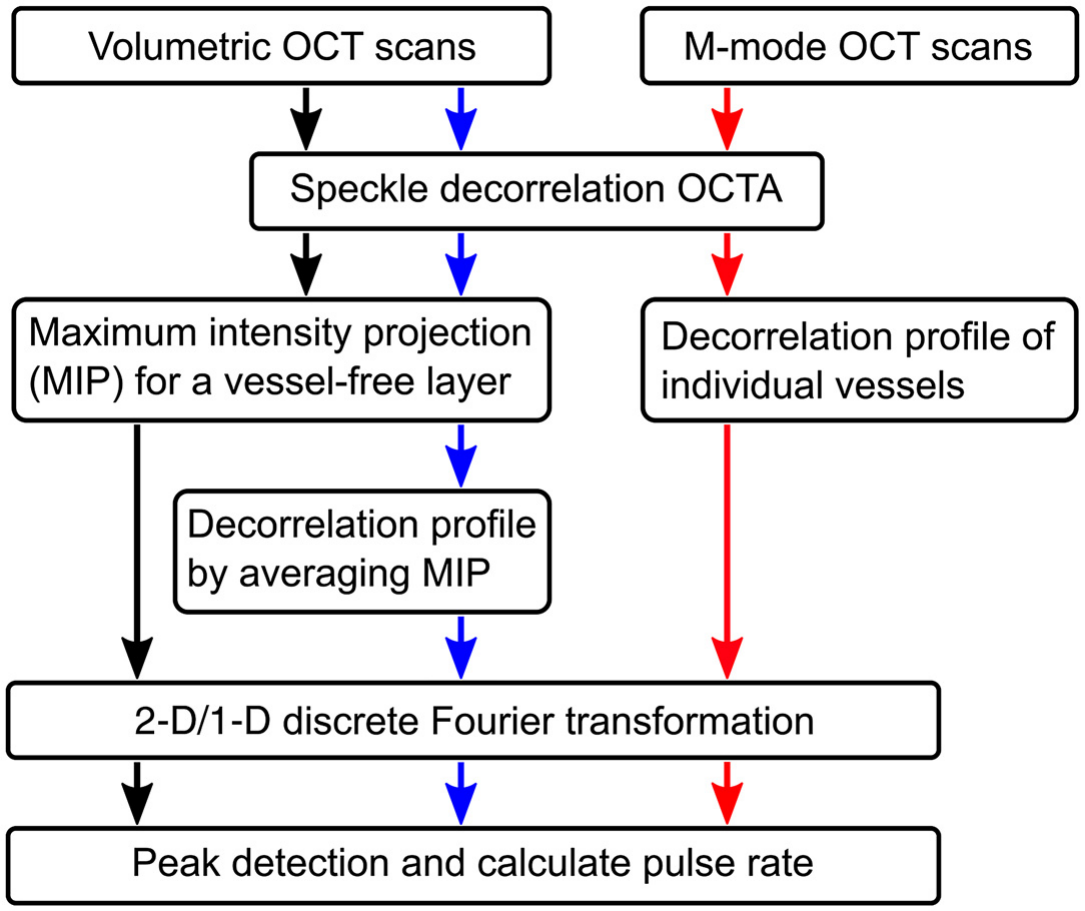
Data processing for pulsatile motion analysis of vessel-free tissue (black and blue arrows) and individual microvessels (red arrows). For vessel-free tissue, detection of pulsatility is obtained either by 2-D FT of vessel-free MIP image or 1-D FT of speckle decorrelation temporal profile that is obtained by averaging the vessel-free MIP image along fast-scanning axis (i.e., x direction).

Figure 1 summarizes the data analysis flows for detection of pulsatility in vessel-free tissue (black and blue arrows) and in the individual microvessels (red arrows). To visualize the pulsatile motion in vessel-free tissue, a second projection depth range was used to generate another MIP image for each weighted OCTA volume, specifically to capture the pulsatile tissue motion-induced OCTA signal independent of the spatial distribution of the vessels. This range was set from the surface to somewhere in the range 50- to 150-μm deep, and mainly located in the avascular epidermis.

In the MIPs from vessel-free tissue, the two axes along the fast (x) and slow (y) scanning directions were converted from the typically used length scale into the time scale based on the scanning speed and the numbers of A-scans and B-scans. A two-dimensional (2-D) discrete Fourier transformation (FT) was then performed on the MIP image data to extract the 2-D frequency spectrum (black arrows in Fig. 1). The sampling time interval and total time along the slow scanning axis were, respectively, 35.6 ms and 21.4 s, resulting in a total detectable frequency range of ±14  Hz with a frequency interval of 0.047 Hz. In contrast, the fast scanning axis corresponded to a total frequency range of ±38  kHz and a frequency interval of 75 Hz (13.2-μs sampling time interval and 13.5-ms sampling total time). Therefore, the pulsatile signature (∼1 to 1.7 Hz for a normal resting heart rate of 60 to 100/min) was mainly captured along the slow scanning axis. Alternatively, the MIP image was averaged along the fast scanning axis. This led to a one-dimensional (1-D) pulsatile motion profile as a function of the time scale along the slow scanning axis (blue arrows in Fig. 1), with the almost negligible variation along the fast scanning time scale. A 1-D discrete FT was performed on this profile to extract the frequency spectrum, which was similar to that generated along the slow scanning axis in the 2-D scenario. The peak in the spectrum corresponding to the pulsatile motion was identified to calculate the pulse rate, which was further validated against the reading from the pulse oximeter.

#### 2.3.2 Analysis of pulsatility in individual microvessels

To detect pulsatility in individual microvessels, M-mode scans were first processed (indicated by the red arrows in Fig. 1) to calculate the speckle decorrelation between each pair of sequential B-scans. The decorrelation was then weighted by the thresholded logarithmic OCT signal as already described. After detection of the tissue surface, a MIP of the weighted speckle decorrelation was taken for the tissue depth range encompassing the blood vessels, revealing the scanned blood vessels. The cross sections of the vessels were manually identified using the OCTA B-scans and the mean OCTA signal in the same vessel cross sections was calculated in each B-scan. This built a decorrelation temporal profile to represent the pulsatile motion of the scatterers in the blood flow, similar to that in the analysis of the volumetric scans (blue arrows in Fig. 1). Therefore, the same processing by FT and peak detection was performed to identify the peak frequency corresponding to pulsatile motion and to calculate the pulse rate. The resulting pulse rates from both volumetric and M-mode scans were then compared to the readings from the pulse oximeter recorded during the OCT scanning.

## 3 Results

Results from detection of pulsatility in vessel-free tissue and in the individual microvessels are presented in Secs. 3.1 and 3.2, respectively. Section 3.3 presents a comparison of pulse rates deduced from all scans with those from the pulse oximeter.

### 3.1 Detection of Pulsatility in Vessel-Free Tissue

**Fig. 2:**
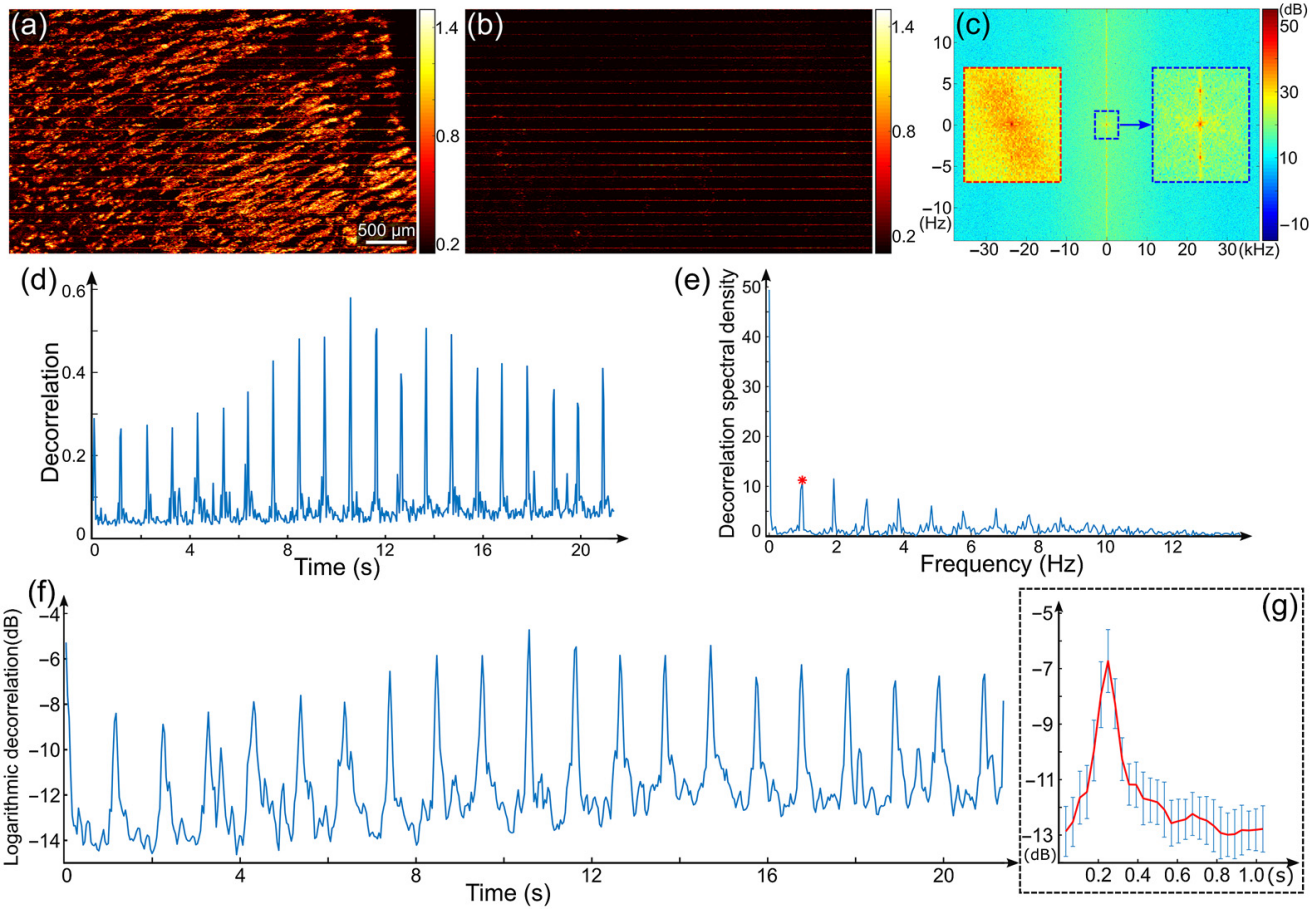
Detection of pulsatility in the dorsal finger skin adjacent to the proximal nail fold in noncontact imaging mode. (a), (b) MIP of decorrelation from skin surface to 400- and 50-μm deep, respectively. (c) Frequency spectrum of (b) with the outlined range (center) magnified in the right inset. The left inset shows the frequency spectrum of (a) in the same frequency range as in the right inset. (d) Decorrelation temporal profile by averaging the MIP in (b) along the horizontal axis. (e) Frequency spectrum of (d) with the marked component corresponding to the pulsatility (red asterisk). (f) Logarithmic decorrelation temporal profile with an averaged period in (g) (scale in decibels).

To analyze pulsatility in vessel-free tissue, we performed noncontact imaging first under the scenario in which the pulsatile tissue motion is dominant. Figure 2 shows the results for 2-D and 1-D FT of the OCTA MIP for a vessel-free layer. Imaging was performed on the dorsal finger skin, adjacent to the proximal nail fold of a 33-year-old subject. The MIP image of the blood vessels in Fig. 2(a) from the skin surface to 400-μm deep shows the local effect of tissue bulk motion as the thin horizontal lines, overlaid on the vessels. These lines in the MIP image of the vessel-free layer (skin surface to 50-μm deep) in Fig. 2(b) present a periodic distribution along the slow scanning (vertical) axis, indicating the correspondence with pulsatile tissue motion. The 2-D discrete FT of the decorrelation in Fig. 2(b) then highlights this distribution in the frequency spectrum along the slow scanning axis in Fig. 2(c). In particular, the magnified spectrum (right inset) of the outlined region in the center of Fig. 2(c) shows local maxima corresponding to the pulsatile motion at ±0.98  Hz, adjacent to the center dc (i.e., zero frequency) component. The capability to resolve this frequency is significantly reduced when blood vessels are included in the MIP for 2-D FT and introduce confounding spatial frequency components produced by the vessel structures. This is shown in the left inset in Fig. 2(c), from the same frequency range as in the right inset but computed using the MIP image in Fig. 2(a). The heart rate inferred from the pulsatile motion (59/min) in the vessel-free tissue is very close to the average rate from the contralaterally located pulse oximeter (60/min), supporting the assertion that the OCTA measurement corresponds to pulsatile hemodynamics.

Alternatively, the processing flow in the middle column in Fig. 1 (blue arrows) was used to average the MIP of the vessel-free layer along the fast scanning (horizontal) axis to generate an enhanced 1-D pulsatile motion profile in Fig. 2(d). 1-D discrete FT then produces the frequency spectrum in Fig. 2(e), indicating the peak corresponding to the pulsatility (red asterisk) at 0.98 Hz. This is in good agreement with both the 2-D analysis and pulse oximeter measurement. This OCT scan displays clear evidence of periodic (pulsatile) motion, leading to the well-defined harmonics in the frequency spectrum in Fig. 2(e). The profile in Fig. 2(d) has a large dynamic range, which can be reduced by taking the logarithm, as in Fig. 2(f). The pulses in different cycles were further averaged to generate a single decorrelation temporal profile to represent the average pulsatile motion over one period, shown in Fig. 2(g).

**Fig. 3:**
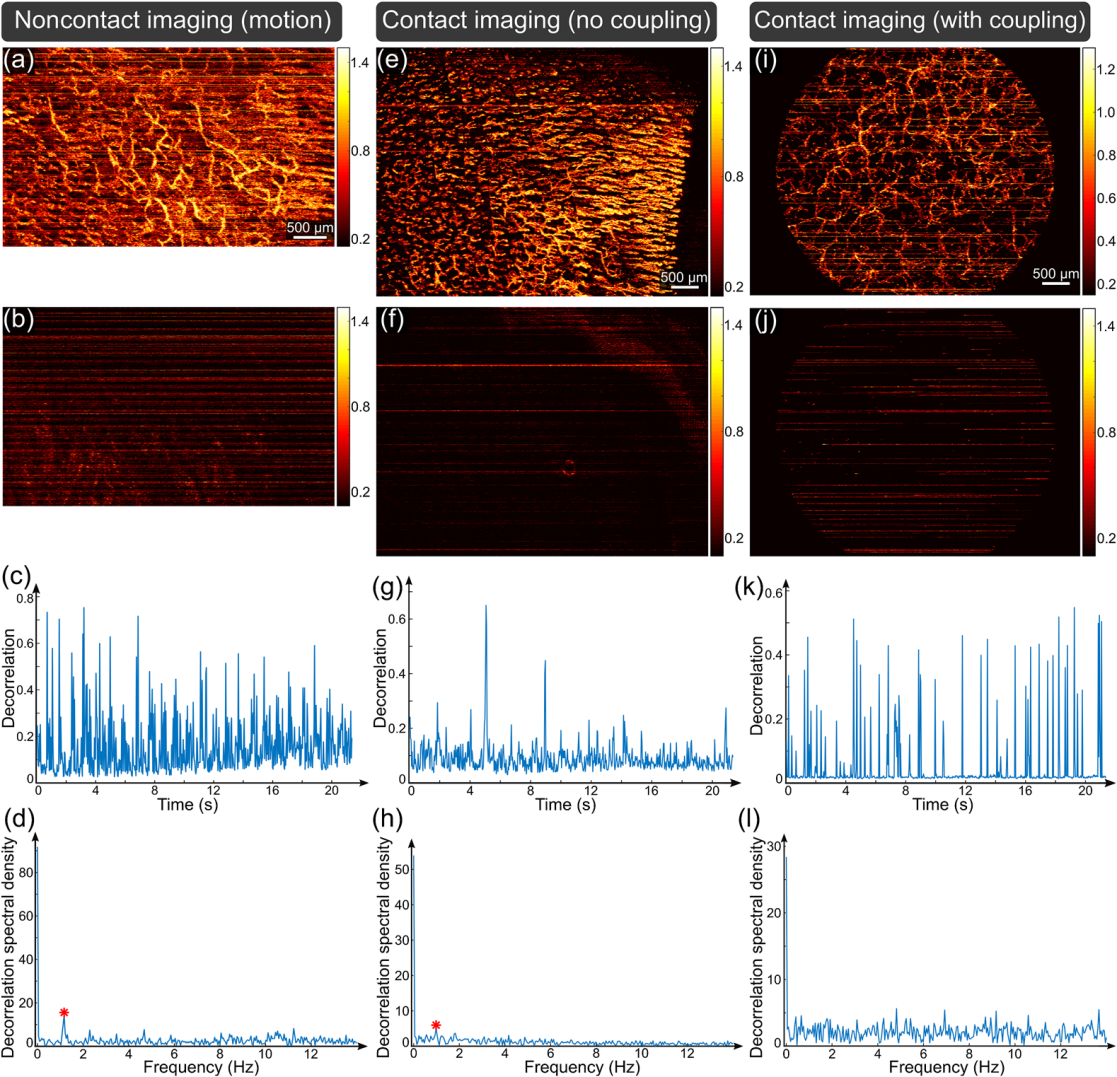
Detection of pulsatility in vessel-free tissue: (a)–(d) from the dorsal finger skin adjacent to the proximal nail fold in noncontact imaging mode with confounding motion artifacts; (e)–(h) from the dorsal finger skin adjacent to the proximal nail fold in contact imaging mode without additional mechanical coupling; and (i)–(l) volar forearm skin in contact imaging mode with additional mechanical coupling. (a), (e), and (i): MIP of decorrelation from skin surface to 450-, 450-, and 400-μm deep, respectively. (b), (f), and (j): MIP of decorrelation from skin surface to 50-, 150-, and 75-μm deep, respectively. (c), (g), and (k): Decorrelation temporal profile by averaging the MIP in (b), (f), and (j) along the horizontal axis, respectively. (d), (h), and (l): Frequency spectrum of (c), (g), and (k), respectively, with the marked components corresponding to the pulsatile motion (red asterisks).

Figure 3 shows the results of pulsatility detection in vessel-free tissue for three other skin locations on different subjects. Figures 3(a)–3(d) demonstrate that the proposed method is sensitive to the pulsatile motion even when it is confounded by other sources of motion. The MIP [Fig. 3(a), skin surface to 450-μm deep] from a scan of the dorsal finger (on the same 33-year-old subject as for Fig. 2) using noncontact imaging shows increased motion artifacts (i.e., high OCTA signal in the static tissue between vessels), though many vessels are visible. The elevated level of motion leads to an increased number of horizontal lines in the MIP of the vessel-free tissue (skin surface to 50-μm deep) in Fig. 3(b). Consequently, it is not possible to clearly identify the periodic distribution corresponding to the pulsatile motion from the OCTA decorrelation signature shown in Fig. 3(c). However, the frequency spectrum is still able to reveal the signature of pulsatile motion as the peak (red asterisk) adjacent to the dc component in Fig. 3(d). The peak frequency at 1.17 Hz corresponds to 70/min, which approximately agrees with the averaged reading from the pulse oximeter (73/min).

Figures 3(e)–3(h) shows a case of pulsatile measurement in contact imaging without additional mechanical coupling. The motion reduction due to contact imaging in the scan of the dorsal finger can be observed in the MIP image of the blood vessels (skin surface to 450  μm) as the reduced number and strength of the horizontal lines in Fig. 3(e), as compared to Fig. 3(a). The scan location was close to the proximal nail fold and shows abundant horizontally oriented capillaries in the right-hand region of the MIP. The decreased pulsatile tissue motion was faintly visible in the MIP of the vessel-free layer (skin surface to 150  μm) in Fig. 3(f), as well as in the resulting decorrelation temporal profile in Fig. 3(g). The pulse rate was still measurable from the computed frequency spectrum in Fig. 3(h) as the identifiable peak at 0.98 Hz, demonstrating good agreement with the pulse oximeter measurement (59 versus 58/min).

Figures 3(i)–3(l) show the results of scans of the volar forearm skin from a 30-year-old subject obtained by contact imaging with additional mechanical coupling. This coupling method can attenuate the large motion observed in Fig. 3(a), but moderate/minor residual motion still persisted for this skin location, as evidenced by the prevalence of thin horizontal lines shown in the MIP image (skin surface to 400-μm deep) in Figs. 3(i) and 3(j) (skin surface to 75  μm). However, when the processing method is applied to the decorrelation temporal profile of the vessel-free tissue in Fig. 3(k), the calculated frequency spectrum in Fig. 3(l) does not show the signature of the pulsatile motion, most likely due to attenuation of the motion caused by the strong additional coupling.

The results presented in Figs. 2 and 3 show that, for the detection of pulsatility in vessel-free skin tissue, noncontact imaging considerably outperforms both contact imaging without and with additional strong mechanical coupling.

### 3.2 Detection of Pulsatility in Individual Microvessels

**Fig. 4:**
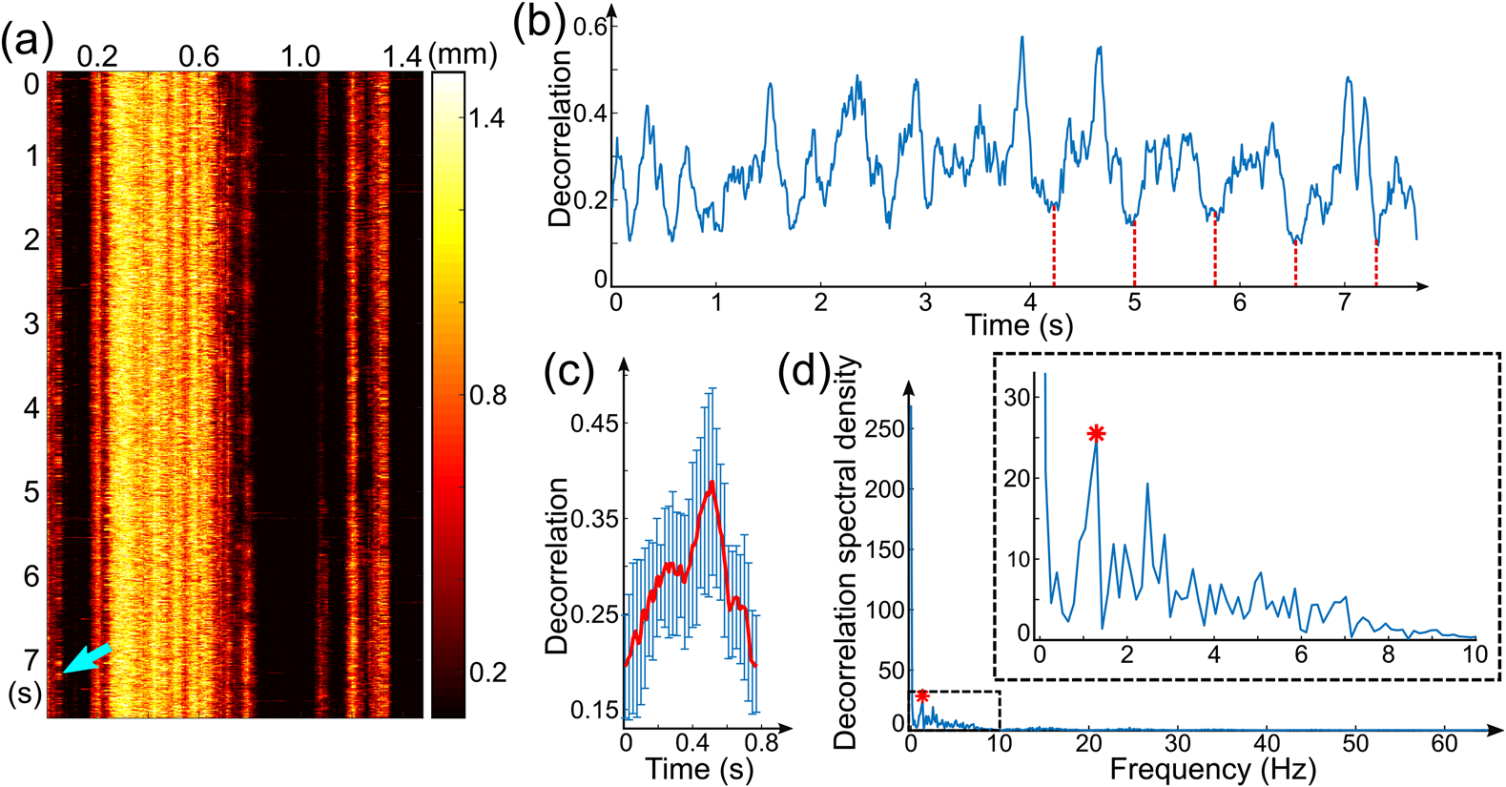
Detection of pulsatility in individual microvessels of forearm skin in contact imaging mode with additional mechanical coupling. (a) MIP of decorrelation from skin surface to 350-μm deep from an M-mode scan. (b) Decorrelation temporal profile of the vessel marked by the cyan arrow in (a). The red dashed lines indicate the equivalent time point in several cycles. (c) Averaged decorrelation temporal profile based on all periods in (b). (d) Frequency spectrum of (b) with the outlined region magnified in the inset. The marked components (red asterisks) correspond to the pulsatile motion of scatterers in the microvessel cross section.

In order to detect pulsatility in individual microvessels, we performed contact imaging with additional mechanical coupling, as this reduces unwanted pulsatile and other bulk tissue motion and enables accurate location and tracking of individual vessels. We extracted the pulsatile motion of the scatterers in the blood flow via the decorrelation signal taken directly from the vessel cross section. Figure 4 shows one such example, measured from the forearm skin of a 30-year-old subject. The MIP image of vessels (skin surface to 350-μm deep) from one M-mode scan is shown in Fig. 4(a), which displays the decorrelation temporal profile over time along the vertical axis and lateral (x) location along the horizontal axis. The cross section of the marked vessel (cyan arrow) is identified from OCTA B-scans. The OCTA signal in the cross section of the vessel was averaged with a window size of 23 μm×10 μm, respectively, in the x and z directions. The averaged decorrelation temporal profile in Fig. 4(b) overall shows a periodic pattern with strong local fluctuations. The dashed lines indicate the equivalent time points in several cycles, which are used to average all cycles, leading to the average decorrelation temporal profile in Fig. 4(c). The calculated frequency spectrum in Fig. 4(d) clearly shows the characteristic frequency peak (red asterisk) corresponding to the pulsatile flow at 1.3 Hz. The resulting pulse rate (78/min) is consistent with the averaged reading from the pulse oximeter (74/min).

The results presented in Fig. 4 show that M-mode acquisition combined with contact imaging utilizing strong coupling is able to detect pulsatility within a microvessel.

### 3.3 Comparison with Pulse Oximetry

**Fig. 5:**
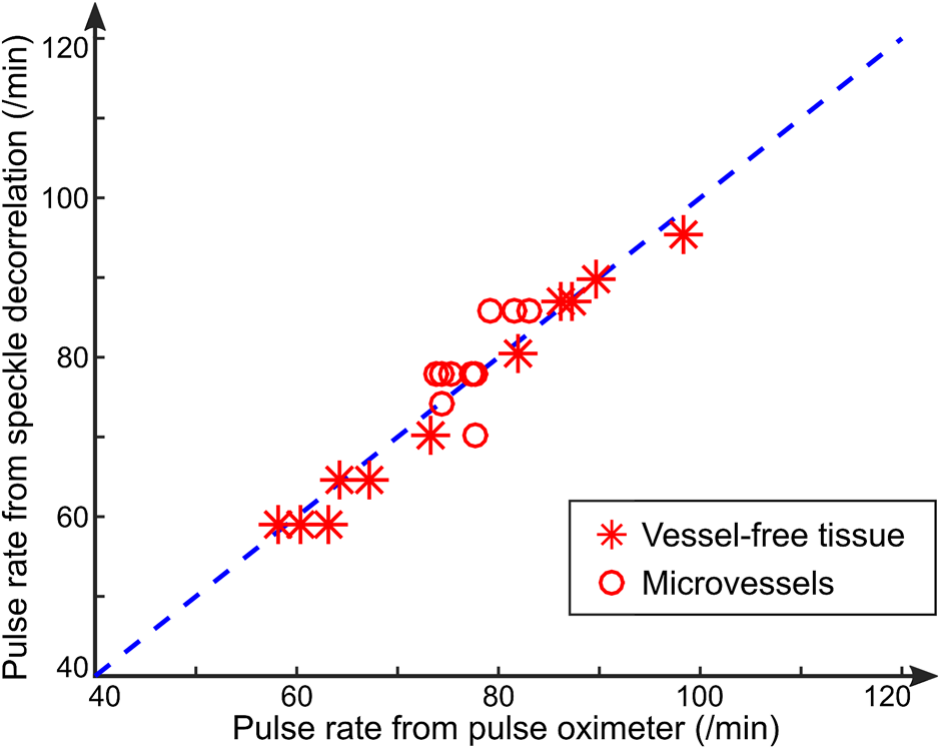
Comparison of the pulse rate measured by speckle decorrelation OCTA and by pulse oximeter. The dashed line indicates equal pulse rates for speckle decorrelation and pulse oximeter. Asterisks and circles are all data points obtained for all subjects and protocols, respectively, from vessel-free tissue and individual microvessels.

Figure 5 summarizes the data obtained on the pulse rate measured from the speckle decorrelation temporal profiles as compared to the averaged pulse rate of the pulse oximeter. Overall, the pulse rate from speckle decorrelation shows good agreement with that from the pulse oximeter (∼3% absolute difference averaged across all data in Fig. 5). Asterisks and circles are, respectively, from all measurements of vessel-free tissue and individual vessels. The good agreement strongly suggests that the pulsatile motion profiles measured from speckle decorrelation of either the vessel-free tissue or the microvessels are, indeed, corresponding to the pulsatility.

## 4 Discussion

**Fig. 6:**
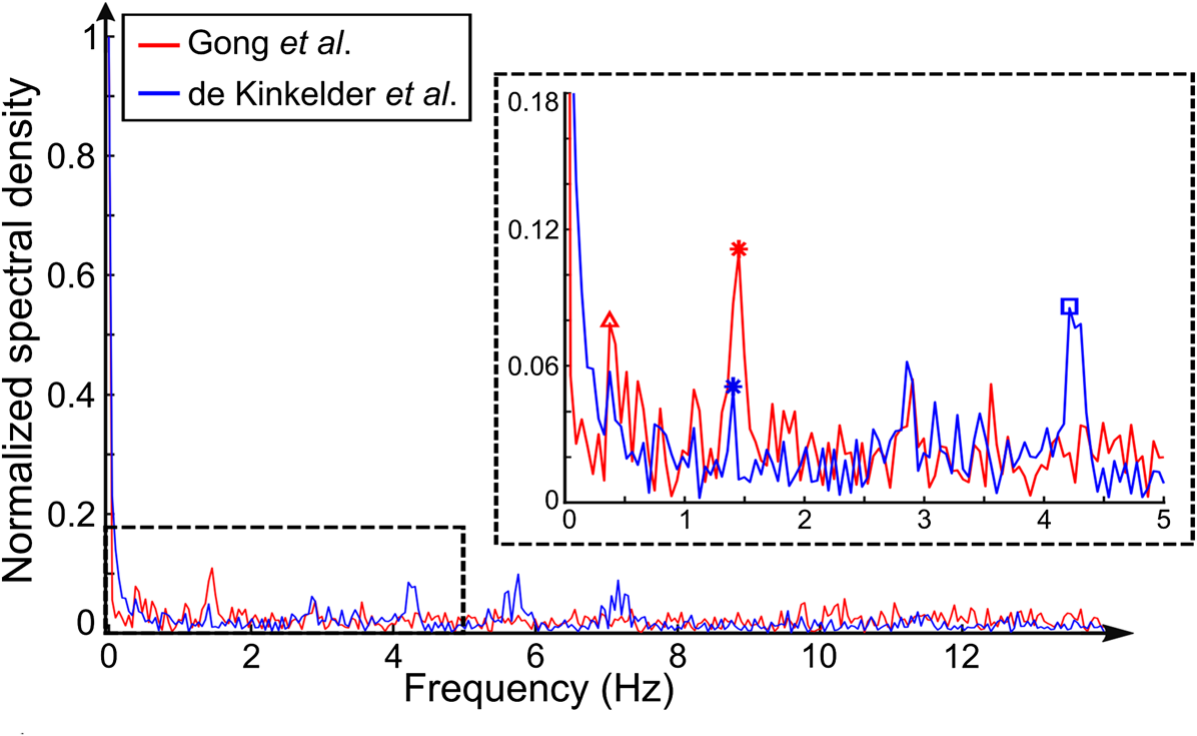
Normalized frequency spectrum of pulsatile motion of the dorsal finger skin adjacent to the proximal nail fold in noncontact imaging (red: our method; blue: de Kinkelder et al.’s method). The outlined region is magnified in the inset. The asterisks and square symbols mark, respectively, the peaks corresponding to the pulsatile motion (first harmonic) and the third harmonic. The peak marked by the triangle likely corresponds to the respiration (22/min).

Although our current method characterizes pulsatility in both individual vessels and vessel-free tissue with speckle decorrelation, others have reported the use of other OCT-derived parameters to detect pulsatile motion in tissue. In particular, de Kinkelder et al.^39^ used the detected shift of the tissue surface along the depth direction in OCT scans of the human retina. We implemented this processing method and compared it to our own and found similar results when the pulsatile motion was clean and dominant, as in Fig. 2. When the pulsatile motion was confounded by other types of motion, our method performed better, as shown in Fig. 6. It shows a stronger peak due to pulsatile motion (red asterisk) with a more accurate pulse rate (87/min) than de Kinkelder et al.’s method (84/min), as compared to the pulse oximeter (average: 87/min). For scans from contact imaging with attenuated tissue motion, our method also showed better performance [e.g., the peaks due to pulsatile motion observed in Fig. 3(h) could not be identified by their method]. The differences are likely due to the sensitivity of speckle decorrelation to pulsatile motion in all directions, as compared to only in the depth direction for Kinkelder et al.’s method. Additionally, the surface detection algorithm may impose a limit on the accuracy in depth (e.g., subpixel motion might not be well detected). The very comparable results in Fig. 6, together with the identification of retinal pulsatility by de Kinkelder et al.’s method, indicate the potential application of our method to retinal imaging, which is the dominant field for OCTA imaging.

Pulsatile blood flow has previously mainly been measured by Doppler OCT, which conveys an advantage over other OCTA variants due to its direct measurement of the flow speed component along the OCT beam propagation direction. It has been applied to the retina, aorta, hind limb, embryo, and trabecular meshwork (for aqueous humor).^26^^–^^34^ In contrast to Doppler OCT, other variants are superior for imaging the microvasculature network, but cannot directly measure the flow speed. In a very recent study on the OCTA variant OMAG, Xie et al.^35^ demonstrated the pulsatile flow signature from individual blood vessels in skin. Consistent with their finding, our evaluation of speckle decorrelation in individual vessels indicated a pulsatile flow signature. We additionally observed the pulsatile motion patterns in vessel-free tissue expected from the pulsatile pressure. Such preliminary work extends pulsatility measurement to OCTA variants beyond Doppler OCT and supplements their well-established capability of imaging the microvasculature network with steps toward a more comprehensive assessment of the microcirculation. We note that our measured pulsatile waveforms present differences to those obtained from Doppler measurements. Although our OCT scans provided the phase signal for Doppler processing, the scanning protocol and parameters did not support a reliable Doppler measurement. Future comparisons to the Doppler method may provide an avenue to understand and calibrate waveforms measured by our method.

We further note that there are many options for the choice of OCTA methods, both intensity-based and complex methods, including speckle variance^21^ and the split-spectrum method^40^ that is used in retinal OCTA. Here, we chose speckle decorrelation for its simplicity, efficiency, and previously shown effectiveness in imaging skin.

The pulsatile motion profiles of scatterers in vessels were observed to be generally irregular [e.g., Fig. 4(b)] and do not routinely exhibit the typical waveform of the pulse oximeter (i.e., a quickly rising primary peak followed by a slowly decreasing secondary peak). The same is true for the pulsatile motion profiles of the vessel-free tissue [e.g., Fig. 2(g)]. The irregular waveform shape may be due to the nonlinear decorrelation versus flow speed relationship.^41^ Previous studies have shown that speckle decorrelation is linear with flow speed only within a small range of flow speeds.^36^^,^^42^ At slow flow speeds, decorrelation is also attributed to Brownian motion; whereas when flow speeds increase sufficiently, the decorrelation signal saturates. In addition, speckle decorrelation is a function of not only the flow speed, but also the OCT signal-to-noise ratio.^41^ All these factors contribute to the noisy pulsatile profiles and the failure of pulsatile motion of scatterers to be detected in several microvessels tested. The latter, however, may also be due to low pulsatility in those microvessels, which could be due to significant pulse reflection upstream in the case of arterioles or due to the detection of postcapillary venules. Our current technique will facilitate the characterization of pulsatility patterns in individual microvessels, which may be useful for identification of anatomy and function. Taken together, our observations suggest future work is needed to characterize the dependence of decorrelation on flow velocity in the context of pulsatility, so as to move toward a more robust measurement of pulsatile motion in blood vessels.

A number of steps might be taken to advance the proposed method toward the full assessment of peripheral microvascular pulsatility in the context of cardiovascular diseases. More practical imaging setups could be developed to better mitigate unwanted bulk tissue motion so that the local pulsatile motion is more clearly captured, as in Fig. 2(f), applicable to a wider range of body locations than explored here. For example, fixation approaches could be adapted based on those used in radiation therapy.^43^

The pulsatile waveform shape (referred to here as the decorrelation temporal profile) should be further studied. To support this, comparisons to alternative methods, such as Doppler OCT or plethysmography, could be made. Care would need to be taken to measure the same local tissue volume at the same time or under the same physiological conditions. Quantification of pulsatile parameters beyond the frequency could be further developed if cleaner waveforms could be produced. In this study, only pulse rate was quantified as it was measurable even when the pulsatile motion was confounded by other types of motion and could be validated against pulse oximetry. With the above completed, the pulsatile motion could be quantified in vessel-free tissue by adopting methods from pulse wave analysis to assess arterial stiffness and central hemodynamics;^44^ whereas the pulsatile motion of scatterers inside microvessels could be used to measure the pulsatility index and resistance index, as performed in Doppler measurements, to allow assessment of local tissue perfusion.^27^

## 5 Conclusion

To conclude, speckle decorrelation OCTA is a promising tool to measure localized pulsatility in peripheral cutaneous tissue. Such a tool has the potential for noninvasive, *in vivo* assessment of the pulsatile hemodynamics with simultaneous visualization of the vessels being assessed. For the detection of pulsatility in vessel-free tissue, we have demonstrated the superiority of noncontact skin imaging. Contact imaging with strong mechanical coupling should be used if detection of pulsatility in individual microvessels is desired. Our demonstrations suggest further validation is warranted to confirm that this tool reflects the physiology proposed here and to fully determine its capacity for assessment of the peripheral microcirculation.
